# Opportunistic Premise Plumbing Pathogens: Increasingly Important Pathogens in Drinking Water

**DOI:** 10.3390/pathogens4020373

**Published:** 2015-06-09

**Authors:** Joseph O. Falkinham, Amy Pruden, Marc Edwards

**Affiliations:** 1 Department of Biological Sciences, Virginia Tech, 5008 Derring Hall, Blacksburg, VA 24060, USA; 2 Via Department of Civil and Environmental Engineering, Virginia Tech, 401 Durham Hall, Blacksburg, VA 24060, USA; E-Mails: apruden@vt.edu (A.P.); edwardsm@vt.edu (M.E.)

**Keywords:** opportunistic premise plumbing pathogens, *Mycobacterium avium*, *Legionella pneumophila*, *Pseudomonas aeruginosa*

## Abstract

Opportunistic premise plumbing pathogens are responsible for a significant number of infections whose origin has been traced to drinking water. These opportunistic pathogens represent an emerging water borne disease problem with a major economic cost of at least $1 billion annually. The common features of this group of waterborne pathogens include: disinfectant-resistance, pipe surface adherence and biofilm formation, growth in amoebae, growth on low organic concentrations, and growth at low oxygen levels. Their emergence is due to the fact that conditions resulting from drinking water treatment select for them. As such, there is a need for novel approaches to reduce exposure to these pathogens. In addition to much-needed research, controls to reduce numbers and human exposure can be instituted independently by utilities and homeowners and hospital- and building-operators.

## 1. Introduction

Opportunistic premise plumbing pathogens are waterborne microorganisms that are normal inhabitants of premise plumbing and cause infections in individuals with predisposing conditions, such as advanced age (>70 years), cancer, or immunodeficiency. Recognized opportunistic premise plumbing pathogens include *Legionella pneumophila*, *Mycobacterium avium* and other nontuberculous mycobacteria (NTM), and *Pseudomonas aeruginosa*. A recently published study showed that the incidence of cases of *Legionella* spp. in the United States was estimated to be 13,000 per year, and that for the nontuberculous mycobacteria was 16,000 annually over the period 2004–2007 [1]. Further, the annual economic cost of those infections in the United States was quite high; namely over $430 million for *Legionella* spp. and $425 million for nontuberculous mycobacterial infections [1]. This is likely an underestimate as the infections reported were only those involving hospitalization and did not include *Legionella* infections that were treated outside of hospitals and also did not include the cost of litigation involved in *Legionella* infections. Unlike the classic waterborne pathogens, such as *Salmonella*, *Shigella*, and *Escherichia coli*, the opportunistic premise plumbing pathogens are native to the premise plumbing environment and ideally adapted to survival, growth, and persistence in drinking water distribution systems and premise plumbing. Premise plumbing has several unique features, such as high surface to volume ratio, unique pipe materials, low organic carbon levels, and periods of stagnation, that select for correspondingly distinct communities of microorganisms. Toward the objective of alerting the drinking water community, we provide a first review of the characteristics of opportunistic premise plumbing pathogens.

## 2. Opportunistic Premise Plumbing Pathogens and Their Public Health Impact

Evidence is emerging that both the number of opportunistic premise plumbing pathogens in drinking water and the number of individuals who are at risk of infection by these pathogens are increasing. Thus, we need to expand our knowledge beyond the limited number of model pathogens reviewed here to others, for example: *Stenotrophomonas*, *Aeromonas*, and *Acinetobacter*. Over the period 2000–2009, the incidence of *Legionella* spp. infections in the United States increased almost 200%, from 0.39 cases per 100,000 persons in 2000 to 1.15 in 2009 [2]. For *P. aeruginosa*, although yearly incidence figures are not available because reporting is not required, a total of almost 11,000 hospital-acquired *P. aeruginosa* bloodstream, pneumonia, and urinary tract infections were identified in the United States over the period of January 1992 through May 1999 [3]. For nontuberculous mycobacteria, both the number of isolates (majority are *Mycobacterium avium*) and cases has increased. Specifically, the frequency of isolation of nontuberculous mycobacteria from clinical specimens in Ontario, Canada has risen from 9.1 to 14.1/100,000 over the period 1997 to 2003; a mean annual increase of 8.4% [4]. Pulmonary nontuberculous mycobacterial hospitalizations increased from 2.1 to 2.4 per 100,000 in Florida; a 3.2% annual increase [5]. As with the numbers for *Legionella* spp. infection, that value is an underestimate as the majority of nontuberculous mycobacteria-infected individuals are not hospitalized. The increase in nontuberculous mycobacterial pulmonary disease is predominantly in women (6.5% per year in Florida and 4.6% in New York) [5].

Increases in both the proportion of older individuals and those that are immunodeficient are major contributors to the increases in opportunistic premise plumbing pathogen infection; the proportion of individuals over 60 years will be 25% by 2025, up from 16.1% in 2000 [6]. The effect of an aging population on infection and disease caused by these waterborne pathogens is illustrated by the 6-fold increased frequency of *M. avium* lung disease in the United States of 15/100,000 in the general population compared to 100/100,000 amongst individuals over 60 years [5]. The same trend holds true for *L. pneumophila* infection [2]. In like fashion, as the number of immunodeficient individuals (e.g., cancer, chemotherapy, and transplantation patients), who are more susceptible to opportunistic pathogen infection, increase, so will the incidence of infection and disease. Changes in building plumbing practices and disinfection emphasizing water conservation might also be increasing the likelihood of the occurrence of these waterborne pathogens and disease [7].

An integrated analysis of the two simultaneous trends of increased opportunistic premise plumbing pathogen occurrence and increased infected individuals is needed in order to identify strategies for better protecting public health now and in the future. It is the objective of this review to: (1) describe the characteristics of opportunistic premise plumbing pathogens that lead to their selection in drinking water systems and premise plumbing (Section 3); (2) identify the challenge of this group of pathogens to the current water treatment paradigm (Section 4); (3) describe methods for expanding knowledge of opportunistic premise plumbing pathogens in premise plumbing (Section 5); and (4) consider how an integrated understanding of the behavior of these waterborne opportunistic pathogens can better inform methods for reducing their numbers in premise plumbing (Section 6). Toward those ends, we present a review of current knowledge of *Legionella pneumophila*, *Mycobacterium avium*, and *Pseudomonas aeruginosa* as exemplars and not as independent, unique waterborne pathogens.

## 3. Selection for Opportunistic Premise Plumbing Pathogens in Drinking Water Systems and Premise Plumbing

Characteristics of opportunistic premise plumbing pathogens contributing to their persistence in drinking water distribution systems and premise plumbing include: (1) disinfectant-resistance, (2) biofilm-formation; (3) survival and growth in phagocytic free-living amoebae; (4) growth at low oxygen concentrations; and (5) ability to grow at low levels of organic carbon. These characteristics mean that these waterborne pathogens are ideally adapted to the unique conditions of premise plumbing. Compared to *E. coli*, the CT_99.9%_ for water-adapted cells of *L. pneumophila* is 1050-fold [8], *M. avium* is 2000-fold [9], and *P. aeruginosa* is 40-fold [10] higher; all three are inherently disinfectant-resistant. Thus, standard disinfectant dosages for drinking water treatment will essentially kill off all but these opportunistic pathogens. Consequently, these relatively slower growing premise plumbing pathogens are able to consume the available organic carbon without competition. Biofilm-formation provides a mechanism to prevent washing out of microorganisms in flowing systems, such as pipes and has been documented for *L. pneumophila* [11], *M. avium* [12], and *P. aeruginosa* [13]. Further, for all three, residence in biofilms substantially increases their resistance to disinfectants [12,13,14]. Resistance to phagocytic killing by free-living amoebae is also a shared characteristic of these opportunistic pathogens. *L. pneumophila* and *M. avium* are not killed, but survive and grow in phagocytic, free-living amoebae [e.g., *Acanthamoeba*, *Vermamoeba* (nee, *Hartmanella*)]; they are amoebae-resisting microorganisms (ARM) [15]. Further, amoebae-grown *L. pneumophila* [16] and *M. avium* [17] are more virulent than cells grown in laboratory medium. In contrast, phagocytosis of *P. aeruginosa* results in rapid killing of *Acanthamoeba castellanii* [18]. Quite possibly, low organic carbon concentrations in drinking water may not prevent the growth of all OPPPs. For example, *M. avium* thrives at assimilable organic concentrations (AOC) as low as 50 µg/L [19]. Further, growth of *M. avium* is stimulated by omnipresent humic and fulvic acids in surface waters [20,21]. *P. aeruginosa* can even grow in distilled water [22]. As it is likely that *L. pneumophila* persists in drinking water systems and premise plumbing in amoebae, rather than as free living cells [15], its growth in water of low organic concentrations is dependent upon growth by phagocytosis of other microorganisms by amoebae.

Periods of stagnation in premise plumbing can increase numbers of opportunistic premise plumbing pathogens, as they are capable of growth even at low oxygen levels, though not under conditions of anaerobiosis. For example, *L. pneumophila* numbers did not fall in hot water heaters when not in use [23], *M. avium* can grow at 6% and 12% oxygen [24] and *P. aeruginosa* can grow in the absence of oxygen using nitrate as a terminal electron acceptor [25]. It might prove to be the case that either high or low oxygen concentrations would lead to the reduction in numbers of these microaerobic bacterial pathogens. High oxygen concentrations were associated with low NTM numbers in surface waters [20,26] and the same holds true for *L. pneumophila* [23].

Taken together, a review of the common characteristics of opportunistic premise plumbing pathogens suggests they are ideally suited for growth in premise plumbing. However, for remediation efforts to be successful in reducing their numbers, the premise plumbing environment must be changed. Opportunistic premise plumbing pathogens survive disinfection and are not washed out of pipes because of biofilm-formation. Phagocytic, free-living amoebae are also not going to reduce their numbers as either the bacteria grow within the amoebae (*i.e.*, *L. pneumophila* and *M. avium*) or kill the amoebae (*i.e.*, *P. aeruginosa*). In the absence of competitors, these waterborne opportunistic pathogens can utilize even the low concentrations of organic carbon to grow, perhaps not even restricted by low oxygen concentrations characteristic of stagnation. The paucity of literature on the response of these pathogens to oxygen levels is a strong motivator for research initiatives.

## 4. Challenge of Opportunistic Premise Plumbing Pathogens to the Current Water Treatment Paradigm

Current water treatment dogma is based on the epic battles of the 20th century, when waterborne pathogens (e.g., *Salmonella*, *Vibrio*) primary originated from fecal contamination of water supplies. Those pathogens are naturally attenuated over the time of exposure to drinking water and by treatment steps including disinfection, because they do not multiply outside of their mammalian host. In contrast, *M. avium* numbers increase with distance from the treatment plant as they multiply in the distribution system [27]. Opportunistic premise plumbing pathogens are not merely transported through pipes, but are actually adapted to growth and persistence in drinking water, especially in building plumbing systems, presenting three challenges to the current paradigm for water treatment. First, source tracking within a water distribution system is meaningless, as the likelihood of amplification and detection increases in the more distal parts of the system. Second, disinfection using the guidance developed for *E. coli*, rather than based on susceptibility of these disinfectant-resistant opportunistic pathogens, can actually select for their predominance. Third, the current location for regulatory compliance sampling, at the treatment plant effluent, is actually where the opportunistic premise plumbing pathogens are least likely to be detected, and utilities do not sample stagnant water in buildings where they are most likely to be detected.

Experience has indicated that it might be impossible to fully eradicate these opportunistic pathogens from premise plumbing in homes, condominiums, apartments, hospitals and office buildings [28,29]. In fact, a recent survey identified detectable levels of *Legionella pneumophila* in about 40% of homes sampled [30]. Another survey showed that 70% of biofilm samples from showerheads collected from homes and institutions across the United States harbored *Mycobacterium* spp. and of those 30% had *M. avium* [31].

Another challenge is that several of the opportunistic waterborne pathogens, including *P. aeruginosa* and *L. pneumophila*, enter a viable, but nonculturable (VBNC) state [32,33]. Cells in the VBNC state are viable but fail to form colonies on agar media; thus standard culture methods will fail to detect them or provide false low numbers for cells [34]. VBNC cells display respiratory activity (e.g., redox dye reduction), membrane integrity (e.g., membrane permeation by dyes), yet can be counted as cells using fluorescent-*in* *situ*-hybridization (FISH) with species-specific 16S rRNA probes or total cells counts [35,36]. The VBNC state is triggered by exposure to stress associated with exposure to: disinfectants, reduced temperature, toxic oxygen metabolites, or starvation [34,35]. Cells of both *L. pneumophila* and *P. aeruginosa* in suspension or in biofilms have been shown to enter the VBNC state and, in that state, the number of colony-forming cells were two-orders of magnitude lower than the total number of cells by FISH [36]. Although colony counts for *M. avium* in drinking water are the same as counts by qPCR [37], there are reports of VBNC in mycobacteria [35]. In addition to detection of the VBNC state, methods have been developed for “resuscitation” of VBNC cells, which include phagocytosis by amoebae [38,39].

## 5. Isolation of Putative Opportunistic Premise Plumbing Pathogens

It is important to recognize that opportunistic premise plumbing pathogenic isolates detected by standardized testing, such as *L. pneumophila* serotype 1, are not necessarily the only pathogenic members of their genera. In fact, because reporting is low or not required in most situations (except for *L. pneumophila*), and most infections go unidentified, these opportunistic plumbing pathogens that we are aware of are likely only the tip of the iceberg. Further, in a practical sense, it would be of value to identify novel opportunistic premise plumbing pathogens to anticipate future emerging waterborne pathogens and associated disease to better inform effective engineering control of waterborne disease. Such an objective requires guidance for detection of emerging premise plumbing pathogens.

Toward that goal, we first identify two emerging pathogens that appear to be members of this group of opportunistic premise plumbing pathogens, yet their source of infection has not been clearly elucidated. Injured U. S. troops in the Middle East were found to be infected by *Acinetobacter baumanii*, a heretofore rarely encountered opportunistic human pathogen [40]. *A. baumanii* has been isolated from drinking water [41], survives and grows in *Acanthamoeba* sp. [42] and is approximately 1000-times more resistant to chlorine than *E. coli* [43]. Another emerging opportunistic pathogen is *Aeromonas hydrophila*. It is found in drinking water [44], forms biofilms [45], and is 50-times more chlorine-resistant than *E. coli* [46].

To detect novel emerging waterborne opportunistic pathogens the following methods for isolation are offered. The approaches are based on the common, shared characteristics of the opportunistic premise plumbing pathogens reviewed above (Section 2). It is probable that a substantial proportion of the microbes isolated will not be pathogenic. However, they will share some characteristics in common with the opportunistic premise pathogens (e.g., disinfectant-resistance, amoebae-resisting, and biofilm-formation). In as much the number of individuals expected to be more susceptible to microbial infections is increasing, it is prudent to be alert to emerging waterborne pathogens. At the outset of this discussion it should be noted that the emerged and new opportunistic premise plumbing pathogens can be in a VBNC state and detection by colony formation might fail [34,35,36]. Consideration should be given to providing “recovery” conditions for cells in the VBNC state, before proceeding with detection based on colony formation on agar media. “Recovery” conditions include incubation in broth media, incubating samples or cells suspensions in toxic oxygen metabolite scavengers (e.g., pyruvate or mannitol), or even incubation in amoebae [34,35,38,39].

### 5.1. Selection for Amoebae-Resisting Microorganisms

One of the mechanisms contributing to the survival and persistence of waterborne bacteria in drinking water distribution systems and premise plumbing is shelter from disinfectant provided by phagocytic amoebae [47]. The most direct way for isolating and identifying such bacterial species (and candidate premise plumbing pathogens) is to recover amoebae and protozoa from drinking water or biofilm samples [15,48,49]. Those methods involve collection of amoebae by low speed centrifugation (1000× *g*), washing the pelleted amoebae free of suspended microbes in the supernatant liquid. Subsequent lysis of the washed amoebae with low concentrations of detergent (e.g., 0.1% Tween 80 or Triton X100) and spread plating on medium suitable for growth should yield colonies resulting from any intracellular bacteria [15,49]. Fortunately, most bacteria are relatively resistant to the low detergent concentrations employed to lyse the amoebae. As many bacterial stains will stain cells within amoebae (e.g., the Gram-stain or the acid-fast stain), amoeba concentrates can be rapidly screened to determine whether they harbor intracellular bacteria before lysis. Employing the approach of first isolating amoebae and then amoebae-resisting microorganisms, methylobacteria, mycobacteria, and Legionella were recovered from a hospital water network [49]. In another study, amoebae in water yielded: *A. baumanii*, *A. hydrophila*, *L. pneumophila*, *Methylobacterium mesophilicum*, *M. avium*, *P. aeruginosa*, and *Stenotrophomonas maltophilia* [15]. This approach also has the added advantage of providing “recovery” conditions for cells in the VBNC state, as has been shown for *L. pneumophila* [39].

### 5.2. Selection for Disinfectant-Resistant Microorganisms

One of the characteristics shared by the opportunistic premise plumbing pathogens is their resistance to disinfectants (e.g., chlorine, chloramine) used in water treatment. As water in some distribution systems and premise plumbing has a residual disinfectant concentration, any surviving microbe in those habitats must be disinfectant-resistant. Amoebae can enhance resistance of resident bacteria, particularly when amoebae are in the cyst form, and protect cells that are not inherently disinfectant-resistant (e.g., *Legionella* spp.) [50]. Disinfectant-residual can not only select for resistant cells, but also diminish numbers of disinfectant-sensitive competitors, allowing plumbing pathogens to proliferate utilizing available organic carbon. Thus, disinfectant-resistance can also be used as a selective measure to isolate premise plumbing pathogens. Microorganisms can be directly selected by adding a disinfectant to a water or biofilm sample at a concentration high enough to kill *E. coli*, but not so high that all microbes are killed (e.g., 1 ppm for 30, 60, and 120 min).

### 5.3. Selection of Biofilm-Forming Microorganisms

Another shared characteristic of opportunistic premise plumbing pathogens is their tendency to adhere to surfaces and proliferate in biofilms. Biofilm-formation is essential for persistence and survival of a microorganism in a flowing pipe system. This is particularly the case for slowly growing opportunistic pathogens such as the mycobacteria and *Legionella*; their growth rates are not high enough to replace cells lost due to liquid flow. Biofilm-formation, signaled by aggregation of cells in culture, may also be a corollary of amoebae-resistance as amoebae phagocytosis is an important factor governing the population composition of biofilms. Coupons that are composed of different pipe materials (e.g., copper, PVC, or stainless steel) can be placed in a water sample for 2–6 h at room temperature and adherent microorganisms can be scraped off or suspended by rapid mixing after gentle washing to remove cells from the water. In this approach, the pipe material coupon acts as a selective agent to pull adherent microorganisms out of suspension with microorganisms displaying different hierarchies of adherence. For example, *M. avium* adherence differs significantly between pipe materials; adherence is highest to galvanized steel, and lowest to glass [51]. In a study of adherence of *L. pneumophila* and *P. aeruginosa* to pipe materials used in premise (“domestic”) plumbing, it was shown that *P. aeruginosa* failed to adhere to copper surfaces but *L. pneumophila* did adhere [36]. The authors ascribed this observation to the reported sensitivity of *P. aeruginosa* to copper ions [36]. Thus it is important to select a surface where one suspects the opportunistic pathogen is in the biofilm.

## 6. Measures to Reduce OPPPs

### 6.1. Introduction to Remediation

There are four levels where remediation measures can be imposed: (i) the water-provider (utility); (ii) a residential building manager; (iii) in a hospital; and (iv) in a single family home, apartment, or condominium. As opportunistic premise plumbing pathogens are rather new, caution should be observed in adopting any remediation measure as they are not based on extensive experience or side-by-side trials, but guided by the characteristics of the microorganisms and factors influencing their presence or absence in drinking water. Further, informing premise owners may be problematic. Although the water, residential building, and hospital industries have existing information transfer networks that would lead to wide dissemination for remediation measures, no such network exists for building managers and homeowners. One solution would be to work through foundations (e.g., NTMir and Cystic Fibrosis Foundations) whose patients are unusually susceptible to infection and disease by opportunistic premise plumbing pathogens.

### 6.2. Remediation by Water Providers

Providers of drinking water (e.g., municipal utilities) could reduce waterborne pathogen numbers by reducing the turbidity of water, as cells of the known plumbing pathogens are relatively hydrophobic and prefer attachment to particulate matter to free suspension in water. For example, turbidity reductions of surface water led to reductions in *M. avium* numbers [27]. Disinfectant residuals should be maintained as waterborne microorganisms can grow in the absence of disinfectants. Evidence that *M. avium*, other NTM and *Legionella* are microaerobic, growing equally well at 12% oxygen and even at 6% oxygen [24] and that NTM numbers are low in waters of high oxygen [20,26], suggests that hyper-oxygenation might reduce *M. avium* numbers and perhaps those of other OPPPs. Further, absence of oxygen has also been associated with *Legionella* die off [52]. Nutrient reduction would be expected to reduce opportunist plumbing pathogen numbers as they require low levels of AOC; for example, *M. avium* requires at least 50 µg AOC/L [19]. Finally, water distribution system pipes could be scoured to reduce biofilms and thereby reduce one source of pathogens; those released from the biofilms.

### 6.3. Remediation by Residential Building Managers

Residential buildings offer more opportunity for infection by waterborne pathogens (e.g., showers via aerosolization) than office buildings. Measures could include: (i) raising hot water heater temperature, as there are lower number of *M. avium* in homes with high hot water heater temperatures [46]; (ii) avoiding recirculating hot water, as microorganisms can grow as they traverse the pipes; (iii) installing single unit hot water systems (perhaps instant heaters) to reduce travel time of hot water; (iv) removing all aerators from taps and faucets, as aerators transfer pathogens to air; (v) cleaning and disinfecting all showerheads, as showerheads have high numbers of waterborne pathogens [31]; (vi) replacing showerheads with large hole, low aeration potential as shower aerosols have high pathogen numbers; (vii), consider building-wide disinfection, as microbes grow in absence of disinfectant; and (viii) increasing aeration of water, as OPPPs may be susceptible to toxic oxygen metabolites.

### 6.4. Remediation in Hospitals

Hospitals and long-term care facilities present special problems as patients or residents are unusually susceptible to infection. In addition to employing all the measures for suggested residential buildings, the following measures could be considered: (i) reduce aerosols through filter entrapment (e.g., micro-trapping or paraffin-coated); (ii) installation of an in-building disinfection system; and (iii) microbial filtration in specific locations (e.g., laboratory, equipment preparation, emergency and operating rooms), where patient trauma will have open routes of infection. Such approaches have been shown to be effective in reducing infections caused by *L. pneumophila* [53], mycobacteria [54], and *P. aeruginosa* [55]. A common element in all those actions was the reduction of microbial-laden aerosols from sources in the hospitals.

### 6.5. Remediation by Homeowners

A number of remedial measures are available to homeowners, including: (i) using a well-water source if possible, as wells have lower numbers of opportunistic pathogens [56] and their numbers increase during travel in utility distribution systems from treatment plant [27]; (ii) raising the hot water heater temperature (>55 °C) as homes with high hot water heater temperatures are associated with lower mycobacterial numbers [53]; (iii) increasing aeration of water might reduce pathogen numbers as a number of opportunistic pathogens are microaerophilic [23,24] and ought to be susceptible to toxic oxygen metabolites; (iv) removing all aerators from taps and faucets, as aerators transfer microorganisms to air; (v) regularly cleaning and disinfecting showerheads; (vi) replacing showerheads with large hole, low aeration potential as showers are sources of pathogen aerosols [31]; (vii) consider installing point-of-use microbiological filters in taps and showers; (viii) using pipe materials that have low biofilm potential, and (ix) reducing the distance from hot water heater to sites of use.

### 6.6. Additional Remediation Measures

Another approach would be to implement ultraviolet (UV) irradiation to kill waterborne microorganisms. Although they are disinfectant-resistant, mycobacteria, *Legionella*, and pseudomonads are no more resistant to UV as are other bacteria. However, implementation of UV-disinfection may cause problems as UV is mutagenic. For example, at the ultraviolet (UV) dosages required to kill 99.9% of *Escherichia coli* cells, the frequency of mutants among survivors is increased 150 to 250-fold [57].

An alternative approach is to consider the microbial populations of premise plumbing and modify the overall community so that opportunistic pathogen numbers are lowered; a probiotic approach [58]. This appears to be an underappreciated action that requires further research and testing. For example, as amoebae serve as amplifying hosts for opportunistic premise plumbing pathogens (perhaps as obligate hosts for growth of *L. pneumophila*), it follows that reduction in numbers of amoebae could result in decreased number of premise plumbing pathogens and hence reduce human exposure. Research ought to be initiated to identify conditions that result in reduction in numbers of amoebae [15] to see whether this action can reduce opportunistic pathogens numbers in premise plumbing. It is also possible that reduced predation associated with reduction in amoebae numbers would lead to increased numbers of total bacteria and waterborne pathogens or damage to plumbing materials [59], so caution must be taken. An extension of that approach would be to identify water quality parameters that encourage colonization, growth, and persistence of biofilm-microorganisms that compete with premise plumbing pathogens for surface adherence and biofilm formation. Examination of data reported in a recent survey of showerhead biofilm samples [31] indicated that the presence of members of the genus *Methylobacterium* was associated with the absence of *Mycobacterium* spp.

## 7. Summary

Evidence is emerging that both the number of opportunistic premise plumbing pathogens in drinking water and the number of individuals who are at risk of opportunistic pathogen infection are increasing. *L. pneumophila, M. avium*, and *P. aeruginosa* as exemplars, but there are likely others related and unrelated that exist and go undetected and unreported. An expanded knowledge of the factors in premise plumbing that select for is needed. These characteristics include biofilm-formation, disinfectant resistance, uptake, survival, and proliferation within an amoeba host. Better defining the characteristics that select for a wide range of opportunistic premise plumbing pathogens, known and unknown, could better guide strategies for effective remediation and control and protect public health.
